# Supplementary material for: Signal requirement for cortical potential of transplantable human neuroepithelial stem cells
Source: Nat Commun. 2022 May 23;13:2844. doi: 10.1038/s41467-022-29839-8 (PMC9126949; doi:10.1038/s41467-022-29839-8)
Supplement: Supplementary file 1 — Supplementary Information [file 41467_2022_29839_MOESM1_ESM.pdf]

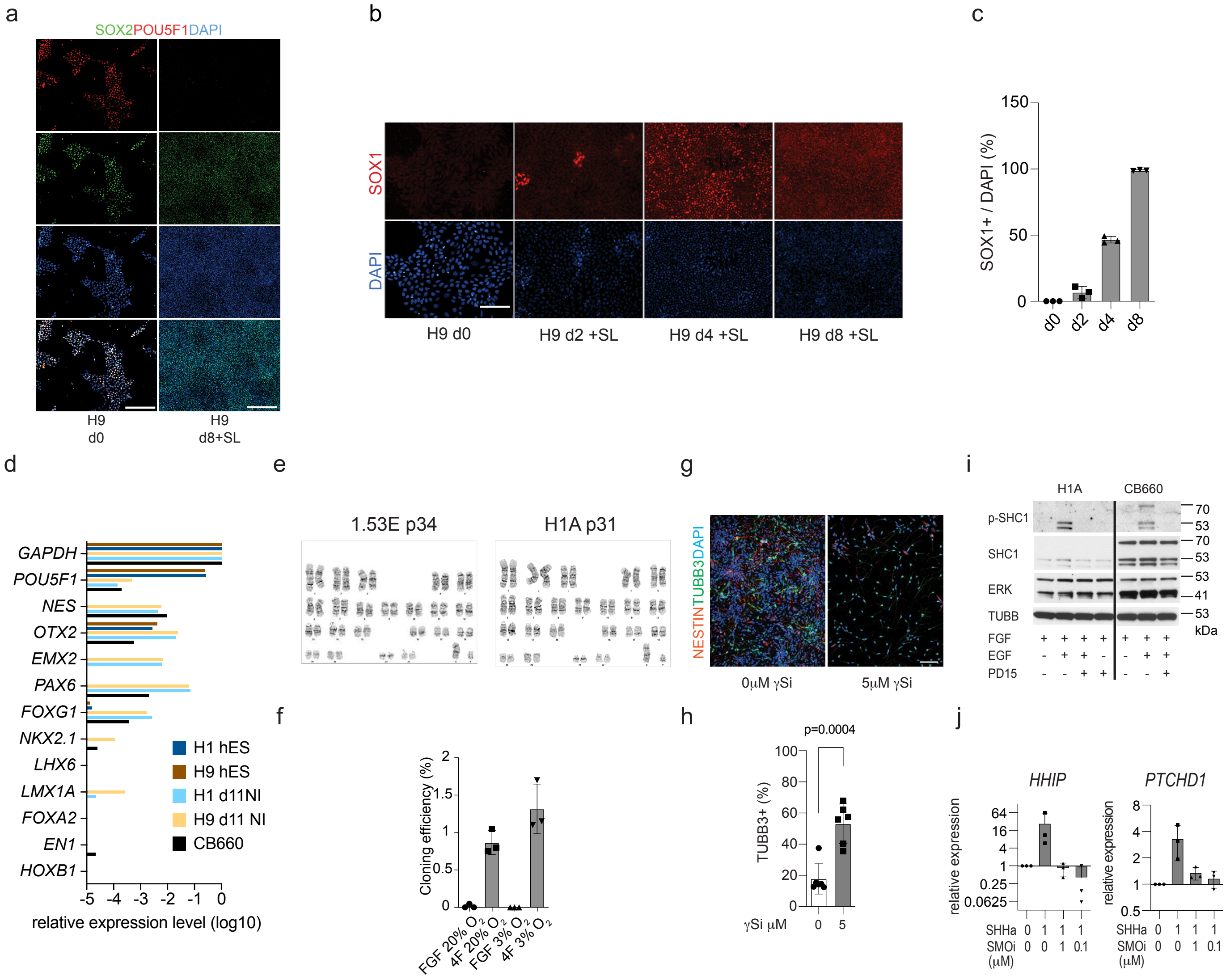

Supplementary Figure 1
Cortical neuroepithelial stem cell characterization

a: Monolayer neural differentiation of H9 hESCs in the presence of SB431542 and LDN193189 (SL) reduced the expression of OCT4 and maintained the expression of SOX2 in day 8 cultures. The experiment was repeated with 3 biologically independent samples. Scalebar:100 μm.
b: The number of neuroepithelium specific SOX1 positive cells increases over 8 days of neural differentiation of H9 hESCs. n=3 biologically independent samples, Scalebar: 50 μm.
c: Quantification of SOX1 positive cells during H9 hESC neural induction, minimum 640 cell nuclei were analysed in 3 technical replicates per time point. (n=3, data are presented as mean±SD)
d: Q-RT-PCR analysis of hPSCs (H1, H9), corresponding day11 neural cultures and CB660 NS cells. Data are presented as mean of technical replicates and normalized to GAPDH.
e: Karyotyping of 1.53E and H1A cNESCs maintained in 4F media show normal chromosome counts.
f: Colony numbers were counted 11 days after plating. (n=3, data are presented as mean±SD)
g: Inhibition of NOTCH signalling with DAPT induced rapid cell cycle exit and neuronal differentiation of cNESCs (H1A) in 11 days in the absence of 4 factors. Immunofluorescent staining of TUBB3 (green) positive post mitotic neurons and NESTIN (red) positive neural progenitors in day 10 cultures. The experiment was repeated with 3 biologically independent samples. scalebar 50μm.
h: Quantification of TUBB3 and DAPI positive cell population. (n=6, data are presented as mean±SEM, two sided Student's T-test).
i: Western blot detection of EGFR activity by SHC1 adaptor protein phosphorylation in control (no EGF), EGFR inhibitor PD15 (PD153035, 2.5μM) treated, EGF treated and EGF plus EGFR inhibitor PD15 (PD153035, 2.5μM) treated H1A cNESCs and CB660 NS cells. The inhibitor validation experiment was not repeated.
j: Hedgehog signalling can be activated in cNESCs (H1A) by SMOOTHENED agonist Purmorphamine (PUR, 1μM) and inhibited by the antagonist Cyclopamine-KAAD (CycK). Activation of SMOOTHENED by PUR upregulates mRNA levels of downstream target genes HHIP and PTCHD1 (n=3, data are presented as mean±SD). Source data are provided as a Source Data file.

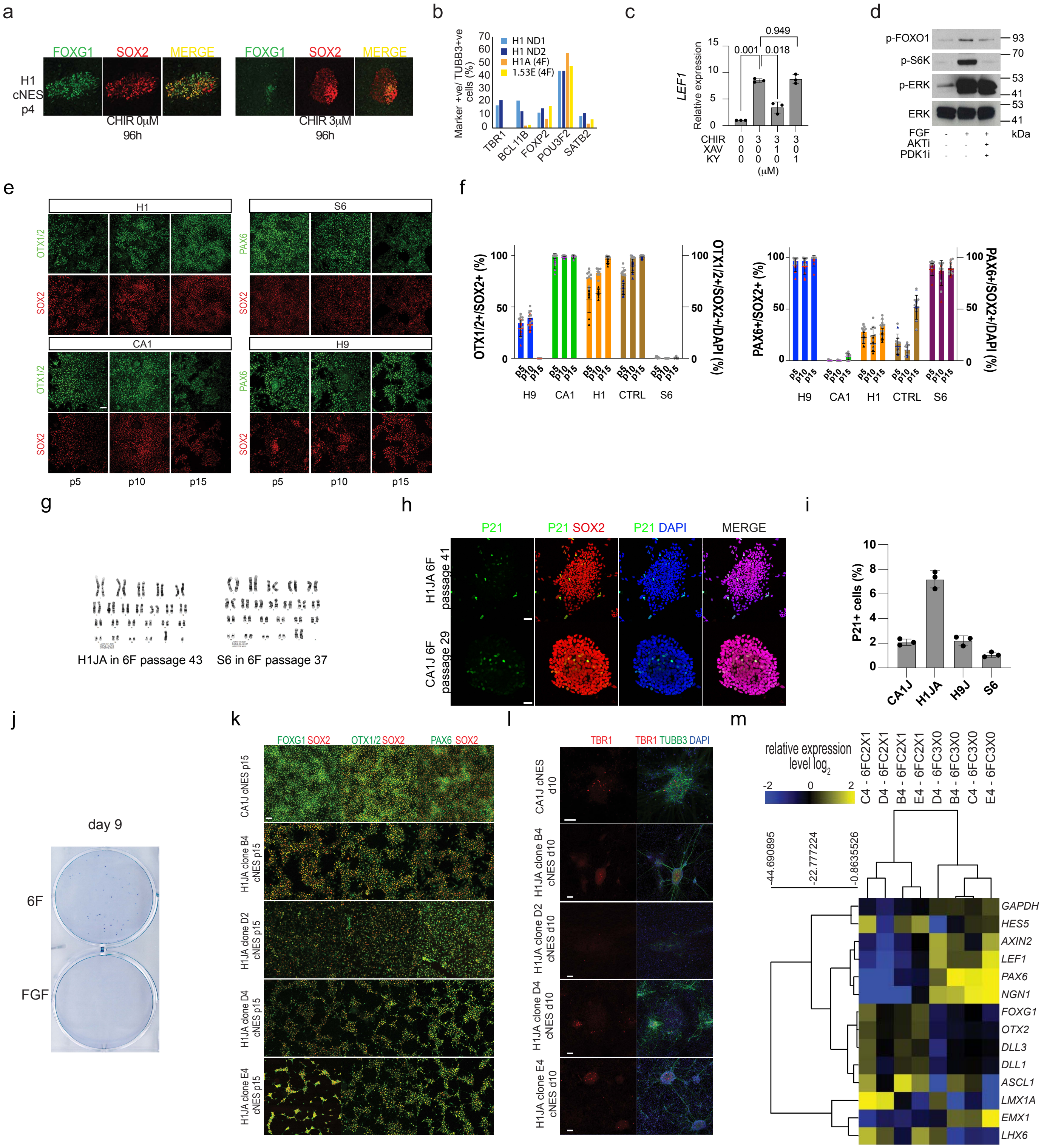

Supplementary Figure 2

cNESCs in 6F media preserve cortical specification

a: Immunofluorescent staining of FOXG1 and SOX2 proteins in passage four cNESCs (H1 PSC derived) after 96 hour treatment with 3μM GSK3 inhibitor (CHIR) or DMSO. The experiment was repeated with 3 biologically independent cell lines.

b: Quantification of deep- (TBR1, CTIP2, FOXP2) and upper-layer (SATB2, BRN2) specific neuronal marker expression in day 31 neuronal cultures of H1A and 1.53E cNESCs maintained in 4F media. Day 70 cultures of H1 hPSCs directly differentiated to cortical neurons without culture in cNESC media were used as control20.

c: Q-RT-PCR comparison of mRNA level of *LEF1* after 12-hour treatment of p0 H1 cNESCs with GSK3 inhibitor (CHIR) alone or combined with either XAV939 (XAV) or KY02111 (KY). (n=3 biologically independent samples, data are presented as means±SD, One-way ANOVA, Dunnett's test)

d: FGF activates both MAPK and PI3K/AKT signalling downstream of FGFR. FGF was withdrawn from cNESCs (H1JA) for 2 hours, cultures were incubated for 10 minutes with inhibitors of AKT activity (AKTi, PDKi) before administration of FGF. Reduction of both phosphorylated FOXO1 and S6K levels after FGF treatment but not phosphorylated ERK levels indicates inhibitor treatment effectively reduced AKT activity in cNESCs. The inhibitor validation experiment was not repeated.

e: cNESCs from 4 independent human PSC line (CA1, H1, S6, H9) were cultured in 6F for 15 passages. Immunofluorescent detection of PAX6 or OTX1/2 in SOX2 positive cNESCs at indicated passages. n=5 biologically independent cell lines. Scalebar: 20 μm.

f: Quantification of OTX1/2, PAX6 and SOX2 positive cNESCs in five independent hPSC derived cNESC lines from multiple passages. Bars show percentage of OTX2 or PAX6 positive NES cells, lines show percentage of OTX2 or PAX6 and SOX2 double positive cells of all cells. n=5 independent cell lines, data are presented as mean±SD

g: Karyotyping of H1JA and S6 cNESCs maintained in 4F media show normal chromosome counts.

h: Quiescent cells were labelled for P21 (Cyclin-dependent kinase inhibitor 1, green) in SOX2 (red) positive cNES cultures maintained in 6F. Passage numbers are indicated for each cell line. The experiment was repeated with 4 biologically independent cell lines. Scalebar: 20 μm

i: Quantification of P21 labelled SOX2+ve cells in 4 independent cNESCs cultures. n=3 biologically independent samples, data are presented as mean±SEM, 6 measurements each. Minimum of 1000 cells were measured for each measurement.

j: Colony formation capacity of H1 cNESCs in 6F media or in FGF alone. cNESCs were cultured in 6F media before plating at low density (200 cells/cm2). Colonies were stained after 9 days with cresyl violet.

k: Immunofluorescent detection of cNESC markers FOXG1, OTX1/2, PAX6 and SOX2 in bulk CA1J and H1JA clonal cNESCs. Inserts show cell nuclei stained with DAPI. The experiment was repeated with 6 biologically independent samples. Scalebar: 20μm.

l: Differentiation of cNESCs from panel K for 10 days with 5μM DAPT. Immunofluorescent detection of TBR1 and TUBB3 positive neurons in cNESCs. The experiment was repeated with 6 biologically independent samples. Scalebar: 50μm.

m: Normalized mRNA levels of genes after ranking by significance of changing after withdrawal of Tankyrase inhibitor XAV939 from 6F media in single cell clones of H1JA cNESC, s. n=4 biologically independent cell lines, colour bar shows log2 level of change from mean calculated from 3 technical replicates. Source data are provided as a Source Data file.

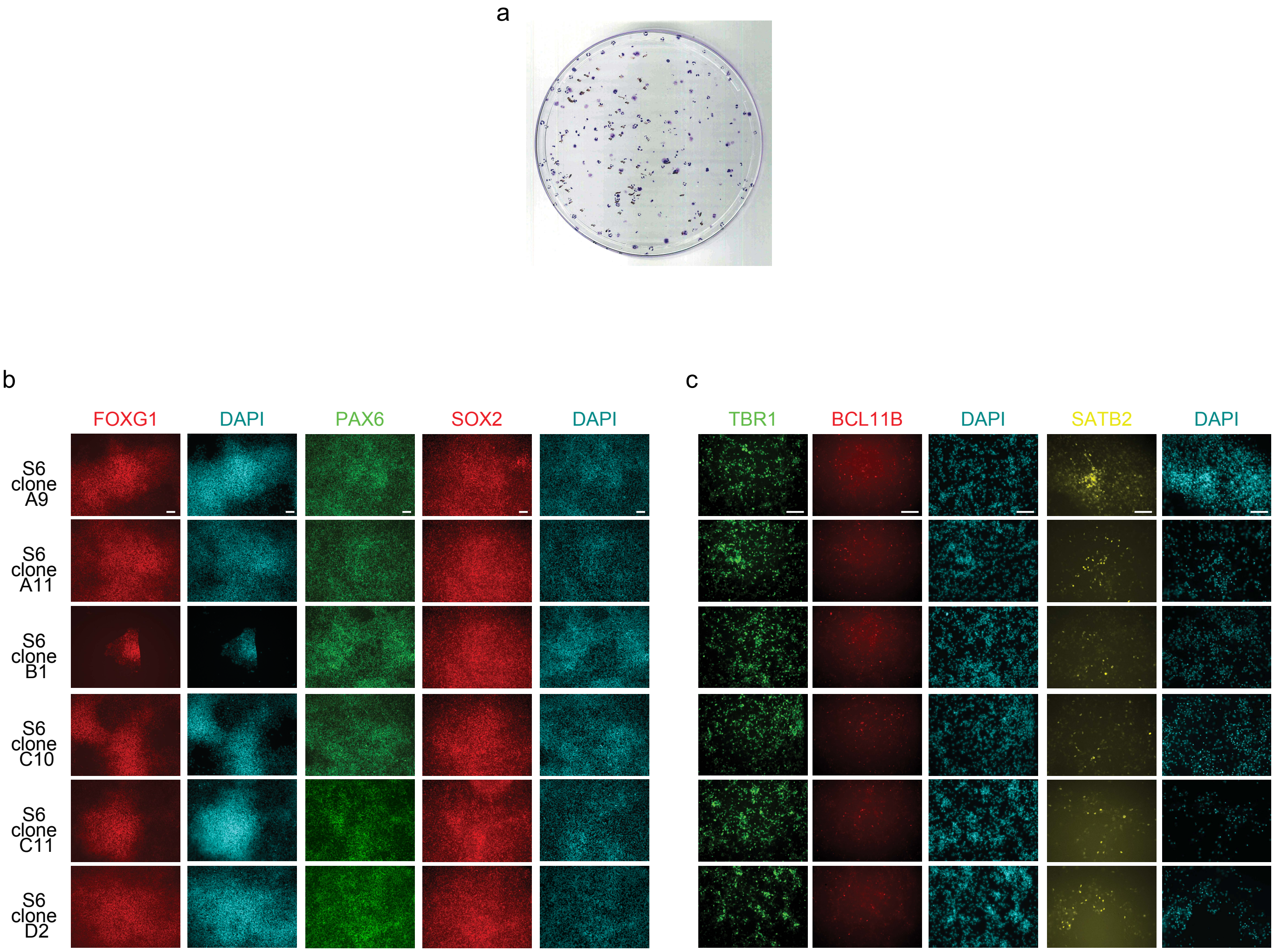

Supplementary Figure 3
Single cell clones of cNESCs

a: Cresyl violet staining of day 12 colonies from S6 passage 9 cNESCs maintained in 6F condition.

b: Examples of immunofluorescent labelling of single cell derived S6 cNESC clones. cNESCs were maintained in 6F condition in 96-wells and labelled for FOXG1, PAX6 and SOX2 dorsal forebrain NES cell markers. Cell nuclei were labelled with DAPI. The experiment was repeated with 45 biologically independent samples. Scalebar: 20µm.

c: Examples of immunofluorescent labelling of deep layer (TBR1, CTIP2) at day 30 and upper layer (SATB2) at day 60 differentiated cultures of S6 cNESC clones in panel b. Cell nuclei were labelled with DAPI. The experiment was repeated with 45 biologically independent samples. Scalebar: 20µm.

a

Cluster	KEGG pathways	GO term enrichment	Genes associated with KEGG pathway
**A**	Glycolysis, Gluconeogenesis, Metabolism	pluripotency<br>monosaccharide metabolic process<br>amino acid transport<br>amine transport<br>hexose metabolic process	*NANOG, POU5F1, GDF3, ZFP42, TDGF1, LDHA, ALDOC, PGAM1, HK2, PFKP, HK1, PCK2, PDHB, GPI, TPI1, ALDH1B1, PGAM4, PGM1, ENO2, PGK1, ENO1*
**B**	DNA replication, Mismatch repair	cell cycle, M phase,<br>chromosome segregation<br>DNA metabolic process<br>DNA repair,<br>response to DNA damage stimulus<br>double-strand break repair	*BRCA1, BRCA2, PCNA, RPA1, LIG1, RPA1, RFC3, POLE3, RFC2, LIG1, POLA1, PCNA, POLA2, MCM2, RNASEH2A, FEN1, MCM6, EXO1, MLH1*
**C**	Phagocytosis, Small cell lung cancer, Terpenoid backbone biosynthesis	neural crest<br>developmentblood vessel development<br>lipid biosynthetic process<br>cell adhesion<br>tube development	*SOX9, CDH11, ETS1, TFAP2B, PAX3, SNAI1, SNAI2, MVD, HMGCR, HMGCS1, FDPS, IDI1, ACAT2, PRKCA, DNM3, PLD1, WASF2, ASAP2, PIP5K1B, ASAP3, VAV2, AKT1, CFL2, MARCKS, PIK3R1, AKT3, PIP4K2B, RXRB, NFKB1, CDK6, RB1, BIRC2, CDK2, LAMA4, CDKN1B, APAF1, FN1*
**D**	No enrichment	No enrichment	-
**E**	Axon guidance	cell projection morphogenesis<br>axonogenesis<br>regulation of neurogenesis<br>cell adhesion<br>cell-cell signaling<br>cell motion	*DCC, PLXNC1, PLXNA3, LRRC4C, EPHB3, EPHB1, PAK7, SEMA5B, PAK2, CXCR4, PAK3, NFAT5, ROBO2, UNC5C, SEMA3A, NFATC1, DPYSL5, NTN1, CDK5, EPHA2, SLIT2, EPHA5, EPHA7, SEMA4G, SEMA6D, SEMA4F, FYN, GSK3B, SRGAP3, SRGAP1*

b

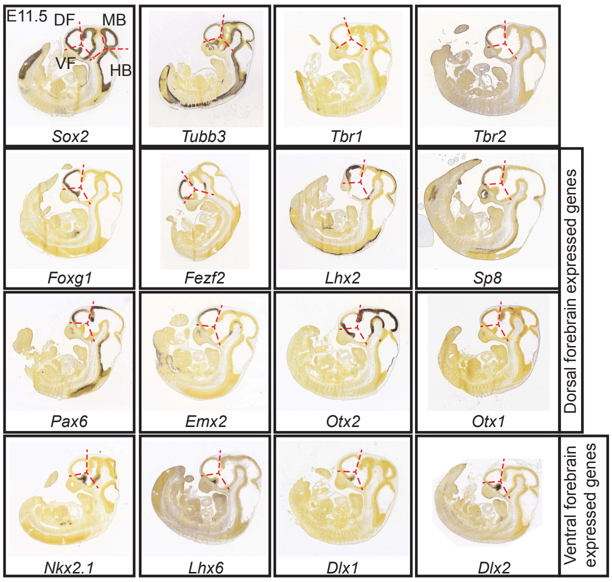

c

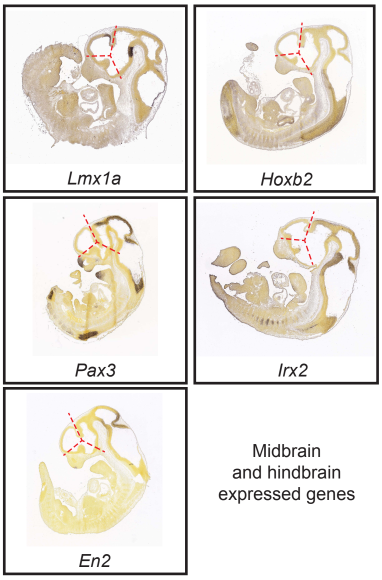

d

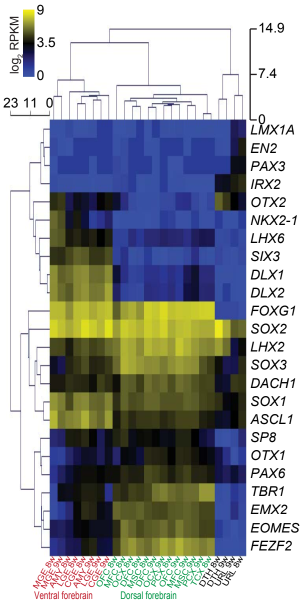

Supplementary Figure 4
Transcriptional analysis of cNESCs and embryonic/fetal brain
a: KEGG pathway and GO-term enrichment of differentially expressed genes in each cluster of genes from Figure 2G.
b: Expression pattern of dorsal and ventral forebrain marker genes from Figure 5e in the E11.5 mouse embryo sagittal sections. Images are from the Allen Developing Mouse Brain Atlas http://developingmouse.brain-map.org/. The developmental age correlates with the differentiation of TBR1 positive deep layer neurons.
c: Expression pattern of midbrain and hindbrain marker genes from Figure 5e in the E11.5 mouse embryo sagittal sections.
d: RNA-seq expression values of region-specific marker genes in the developing 8-9 week old human brain. Normalised RPKM values are from the BrainSpan Atlas of the Developing Human Brain (http://www.brainspan.org/). MGE-medial ganglionic eminence, LGE-lateral ganglionic eminence, CGE- caudal ganglionic eminence, AMY-amygdaloid complex, OFC-orbital frontal cortex, MFC-mediofrontal cortex, OCX-occipital cortex, DFC-dorsolateral prefrontal cortex, MSC-primary motor and sensory cortex, PCX-parietal neocortex, TCX-temporal neocortex, DTH-dorsal thalamus, URL-upper rhombic lip. Source data are provided as a Source Data file.

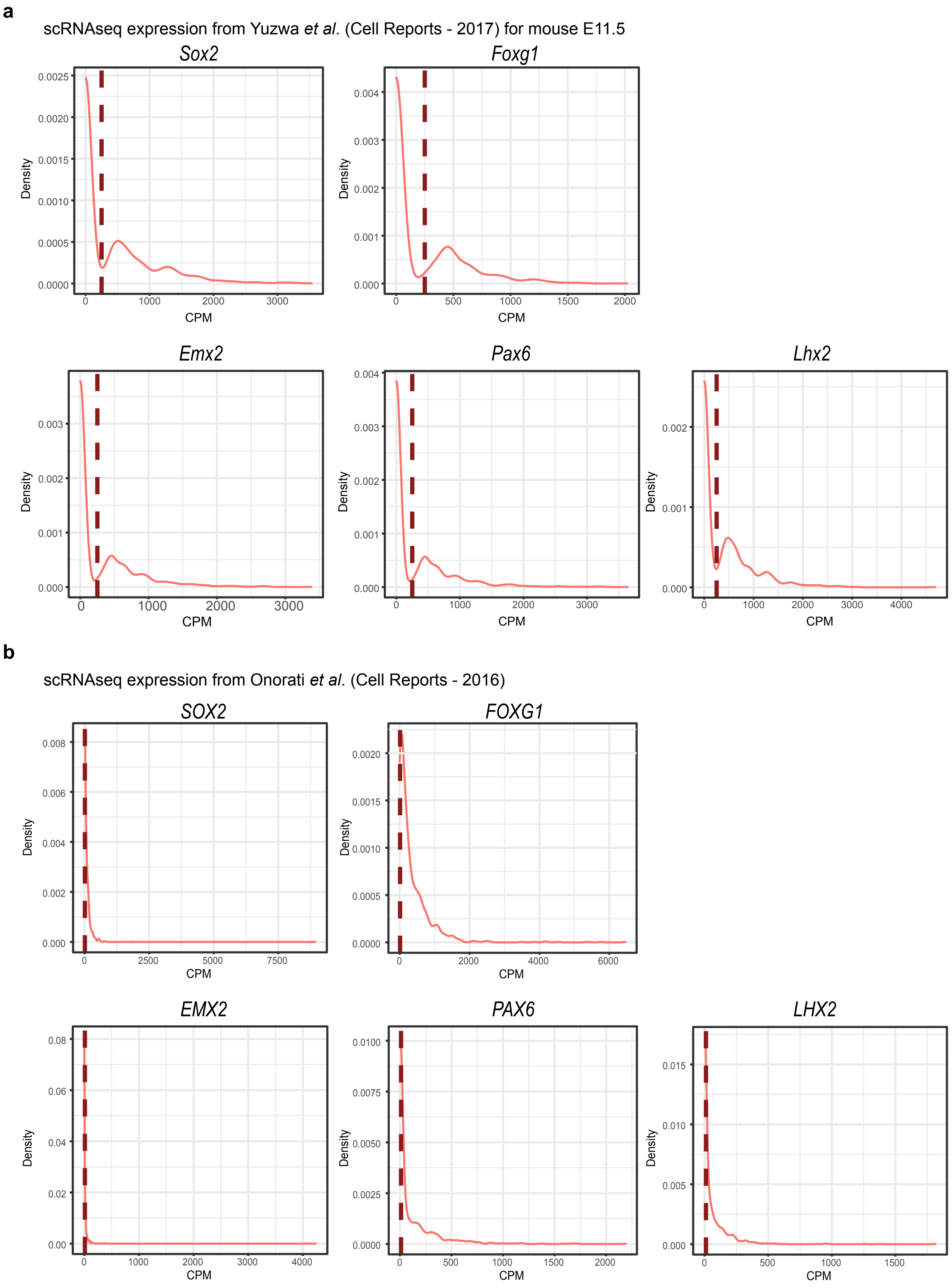

Supplementary Figure 5
scRNA seq expression of cortical markers
Density plots of scRNAseq expression per cell for genes corresponding to in vivo cortical progenitor markers, in a) Yuzwa et al. data 37 at embryonic day 11.5 , b) Onorati et al. data 38 and CPM cutoff for expression is indicated as dashed red line.
Source data are provided as a Source Data file.

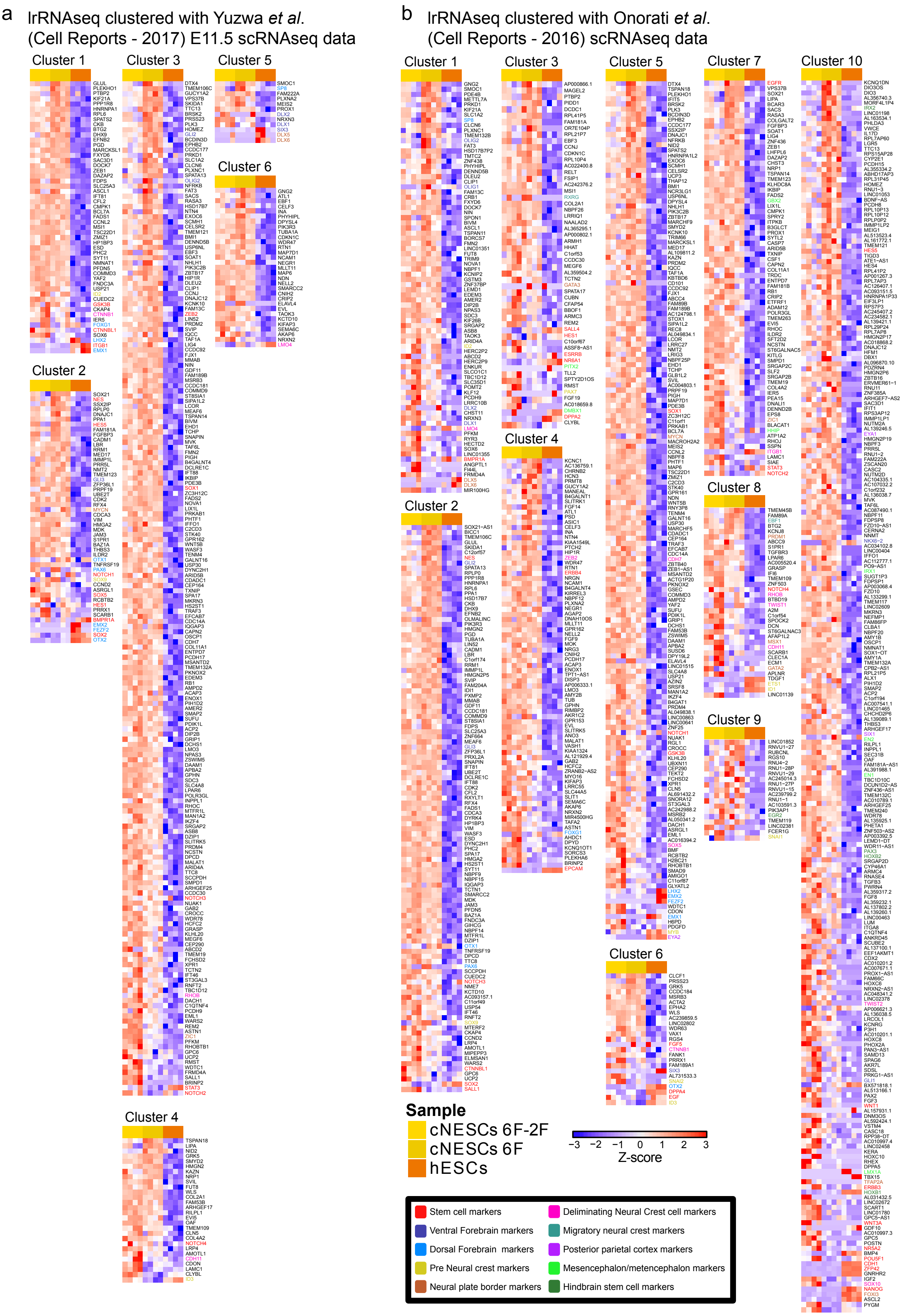

Supplementary Figure 6:
Cluster analysis of cNESCs in 6F and 6F-2F
Gene expression heatmaps (as Z-score of log2(CPM+1)) of lrRNAseq from cNESCs and hESCs, for cNESC marker genes obtained from literature and differential expression analysis. Genes are clustered according to clustering from a) Yuzwa et al. mouse scRNAseq data at embryonic day E11.5 37. b) Onorati et al. human scRNAseq data 38 and marker genes of interest are highlighted in color. Source data are provided as a Source Data file.

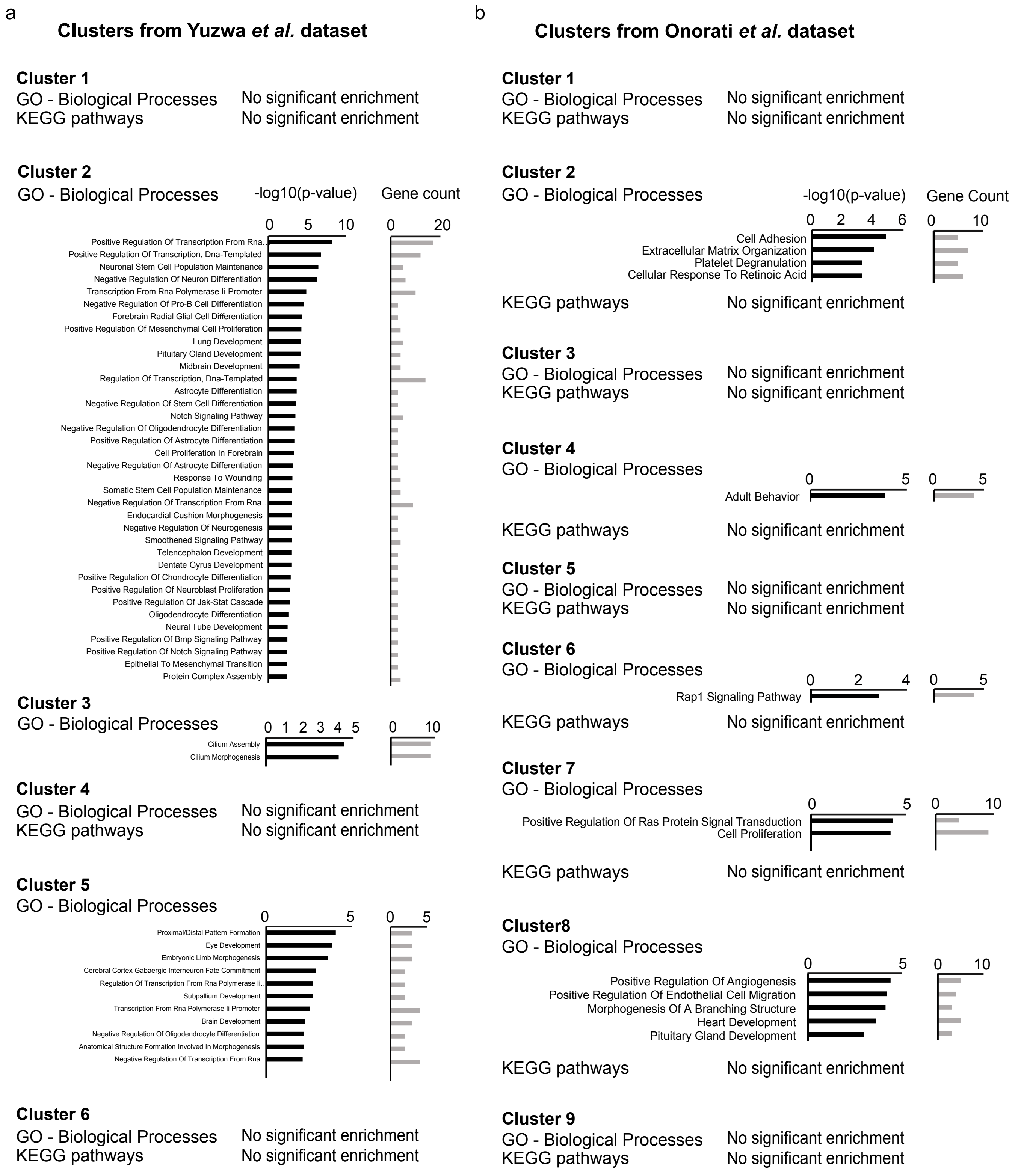

Supplementary Figure 7:
Gene set enrichment of scRNAseq clusters
GO term enrichment analysis for gene clusters obtained from a) Onorati et al. human scRNAseq data 38 and b) Yuzwa et al. mouse scRNAseq data for embryonic day E11.5 37. Full names for GO terms: Positive regulation of transcription from RNA Polymerase II promoter, Negative regulation of transcription from RNA Polymerase II promoter, Regulation of transcription from RNA Polymerase II promoter involved in forebrain neuron fate commitment, Negative regulation of transcription from RNA Polymerase II promoter. Source data are provided as a Source Data file.

a

b

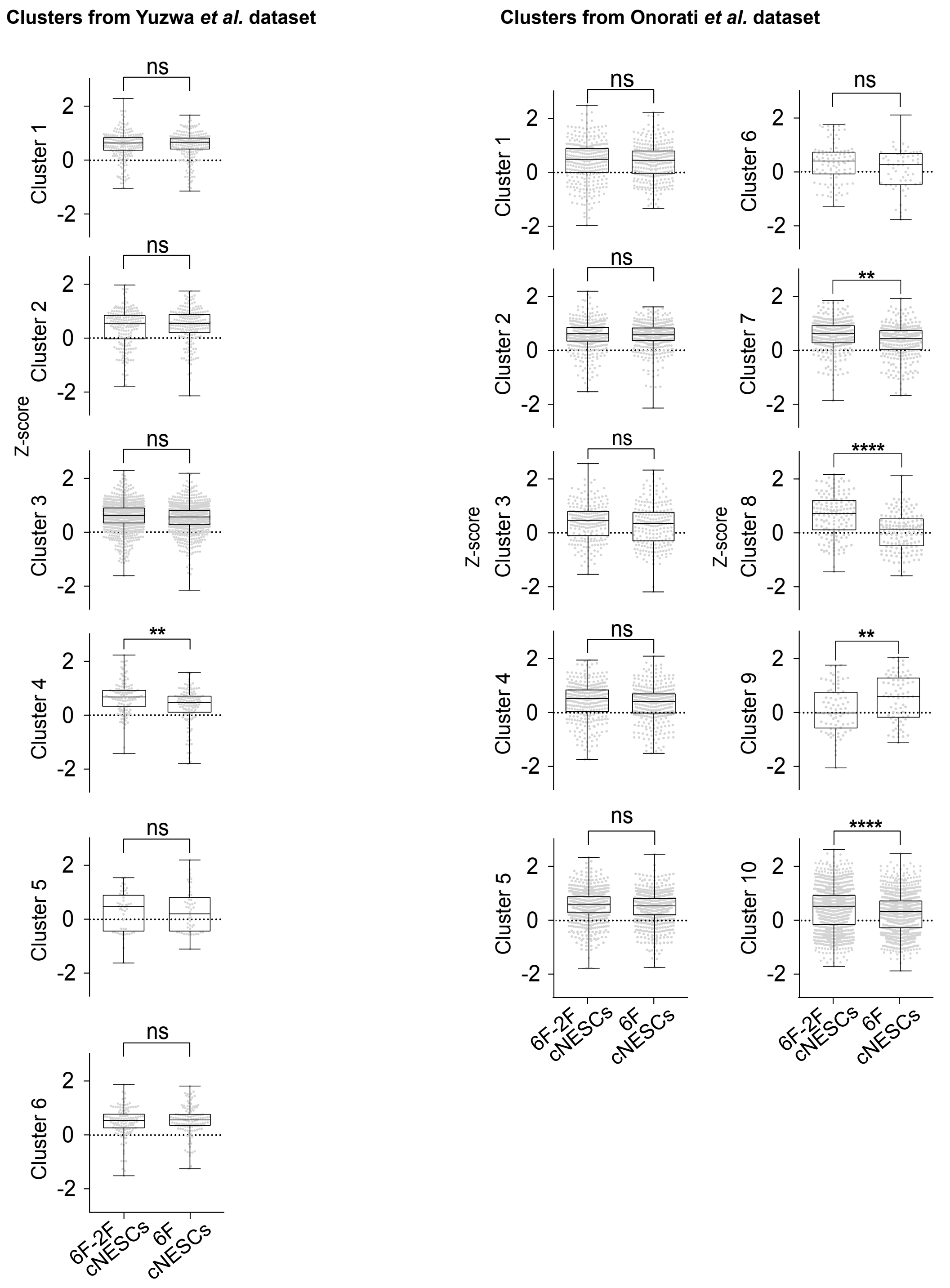

Supplementary Figure 8
Genome wide transcriptional changes after inhibitor switch
Boxplots showing expression (Z-score of log2(CPM+1)) of genes in 4F and 6F cNESCs for gene clusters obtained from a) Yuzwa et al. mouse scRNAseq data for embryonic day E11.5 [37] and b) Onorati et al. human scRNAseq data [38]. Source data are provided as a Source Data file.

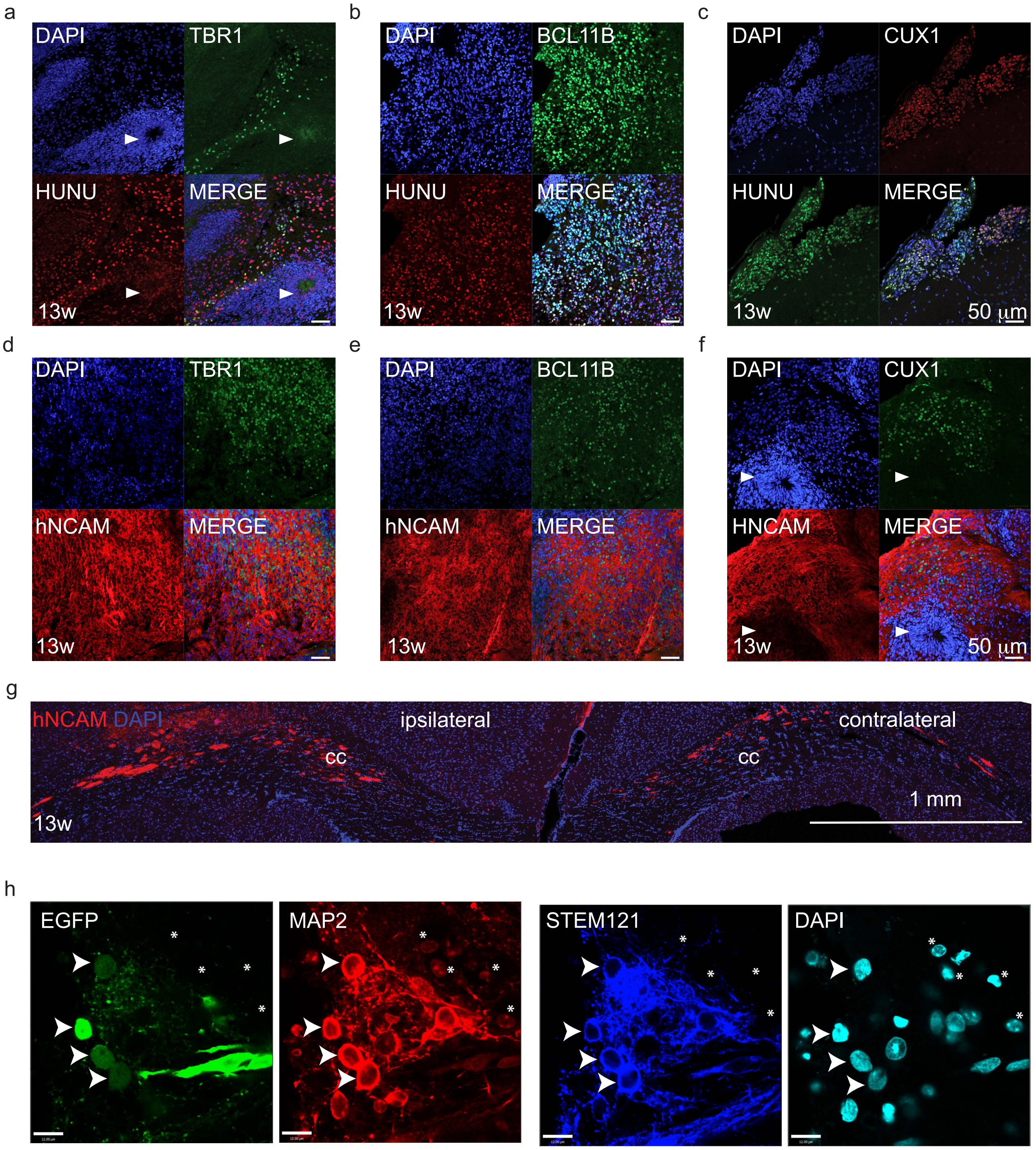

Supplementary Figure 9

Cortical neurons differentiate and mature *in vivo* from human cNESCs

a-f: Transplanted human cNESCs differentiated in situ to TBR1 (a,d), BCL11B (b,e) and CUX1 (c,f) positive HuNu and hNCAM expressing cells by 13 weeks after transplantation. Images are low magnification from Figure 6e-j. Human cNESC rosettes are indicated by arrowheads.

g: Human NCAM positive neuronal neurites were detected in the contralateral hemisphere of transplanted mice after 13 weeks post transplantation. Arrowheads indicate polarised neural rosettes.

h: Immunofluorescent detection of differentiated human cells (H1JA-APCE) in the NSG mouse cortex. Cytoplasmic marker STEM121 (blue) identifies human neurons that express MAP2 (red) and EGFP (green) indicated by arrowheads, triple negative host cells are indicated by astrisks. The experiment was repeated with 3 animals.

Western blot scanned images related to Supplementary Figure 1i

Total ERK
42, 44 kDa

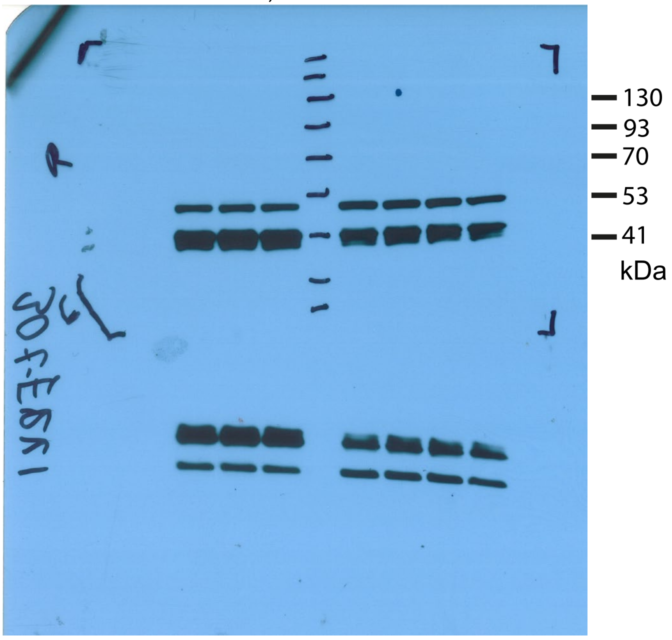

TUBA1B
52kDA

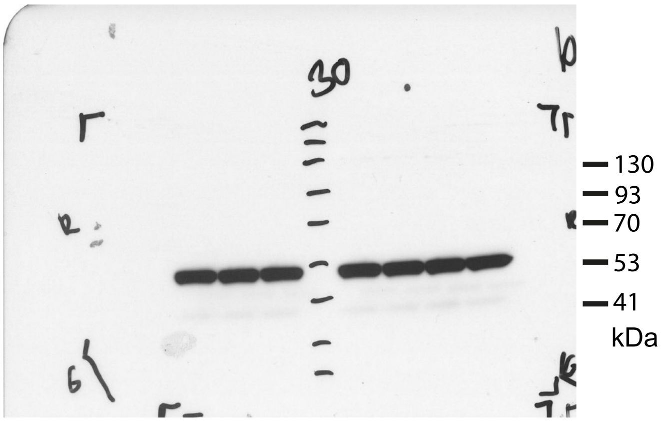

Total SHC1
46, 52, 66 kDa

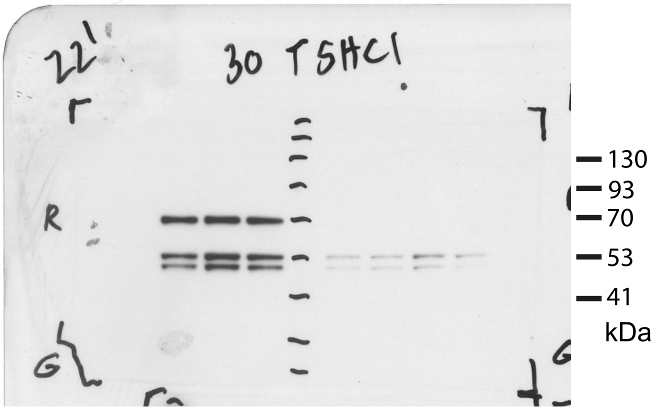

p-SHC1
46, 52, 66 kDa

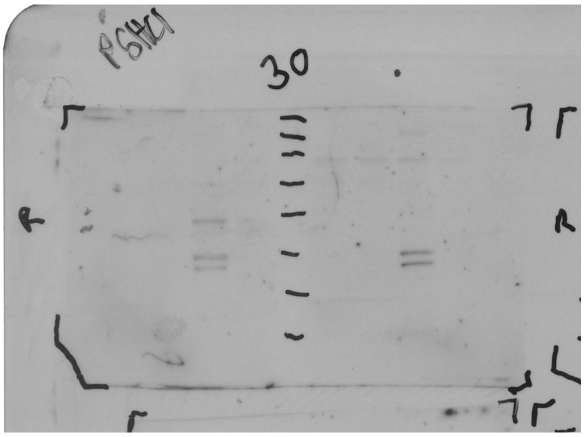

Western blot scanned images related to Supplementary Figure 2d

p-FOXO1
82kDa

p-P70/S6K
70, 85kDa

p-ERK
42, 44kDa

130
93
70
53
41
kDa

p-ERK
42, 44kDa

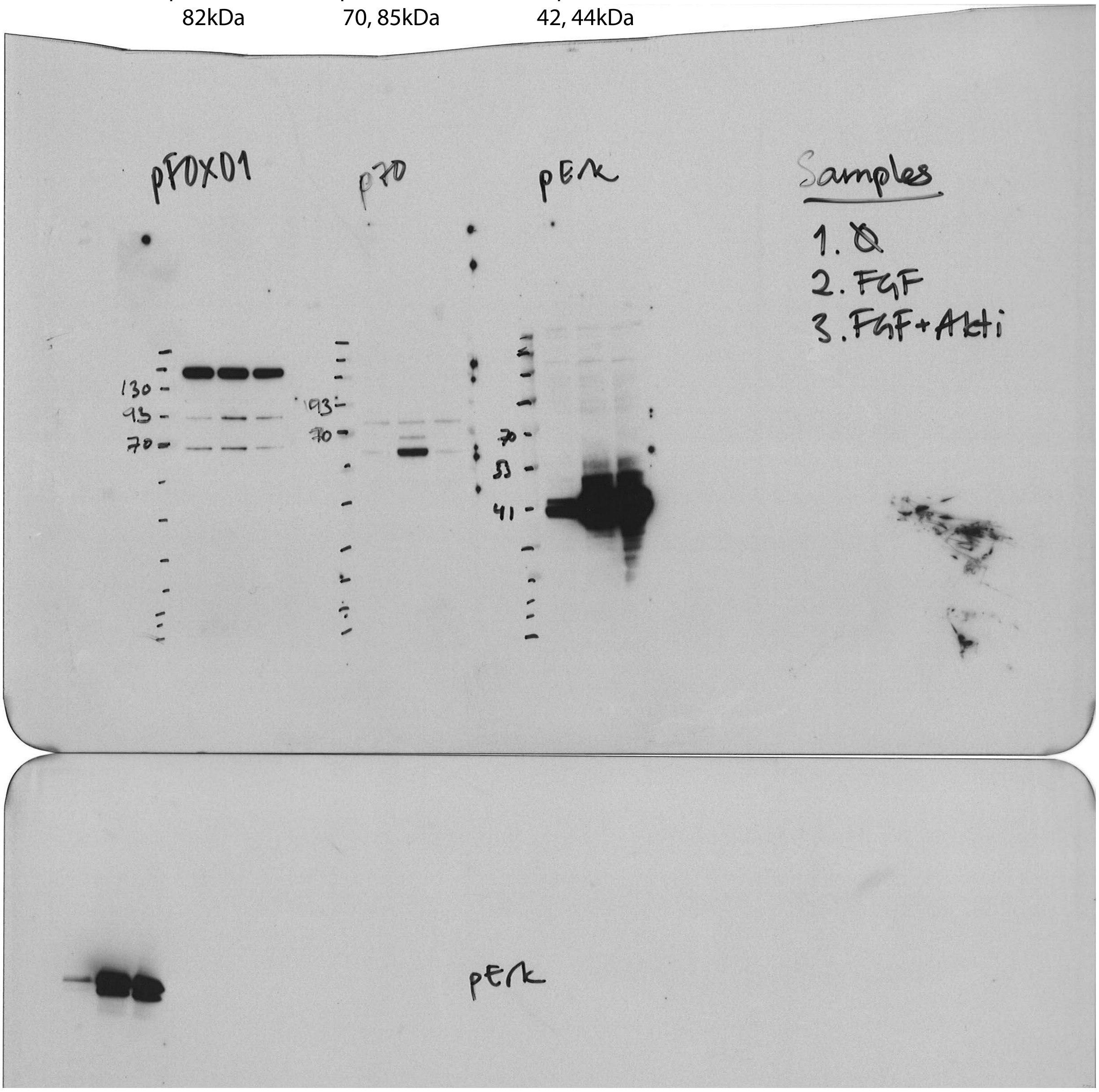

Total ERK
42, 44kDa

130
93
70
53
41
kDa

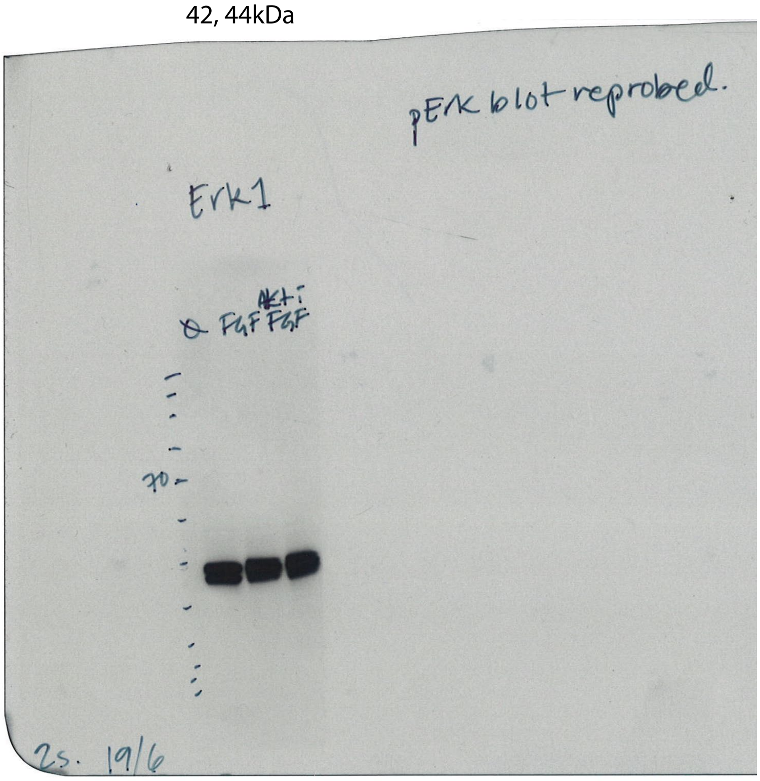

Cortical neural induction protocol with dual SMAD inhibition

NEURAL INDUCTION

1. Culture hPSCs in mTESR1/E8 media feeder free on Geltrex at 37˚C in 5% CO_2
2. Refresh media 15 minutes before cell collection
3. Detach hPSCs with Accutase 5' at 37˚C with tapping or until most cells are floating
4. Centrifuge cells diluted to 10x volume with basal media at 300xg
5. Count cells

6. Plate hPSCs at 50,000-100,000 cells per cm2 in N2B27 media with 10µM SB43, 100nM LDN19 (N2B27 SB/LDN) and 10µM Y27, culture at 37˚C in 5% CO_2
7. Change N2B27 SB/LDN media every second day without Y27 inhibitor
8. on day 8-10 detach NES cells with 500 µM EDTA in PBS 3' at Room Temperature
9. Aspirate EDTA, wash clumps of cells off with DMEM/F12 with 0.1% BSA
10. Centrifuge cells diluted to 5ml at 150xg for 2min
11. Resuspend cell clumps in 6F media (with 10µM ROCK inhibitor in the first 3 passages)
12. Plate cNES cells at 1:1 ratio (approx. 300,000 cells per cm2) on laminin fibronectin coated surface in 6F media containing CHIR99021 2µM and XAV939 1 µM for passage 1

cNESC MAINTENANCE

13. Detach cNES cells in 90-100% confluent cultures with 500 µM EDTA in PBS 1' at Room Temperature
14. Aspirate EDTA, wash clumps of cells off with DMEM/F12 with 0.1% BSA
15. Centrifuge cells diluted to 5ml at 150xg for 2min
16. Resuspend cell clumps in 6F media (with 10µM ROCK inhibitor in the first 3 passages)
17. Plate cNES cell clumps at 1:10 ratio in 6F media
18. Test CHIR99021 3µM and XAV939 between 1-2 µM concentration for each hPSC cell line.

NEURAL DIFFERENTIATION

19. Plate 50-100.000 cells per cm2 on laminin-coated surface in Neural Differentiation I Media
20. Change media every other day for 14 days.
21. On day 14-16, dissociate cells with Accutase and replate as single cells on poly-ornithine coated (1µg/cm2) and laminin-coated glass coverslips at 10-50,000 cells per cm2 in Neural Differentiation II Media.
22. Replace half the media every 2 days.

N2B27 SB/LDN (Neural Induction Media):

1:1 DMEM-F12: Neurobasal
0.5x N2 supplement (with 20µg/ml final Insulin-Zinc concentration) 0.5x B27 supplement with Vitamin A
2mM Glutamax
0.1mM Beta-mercaptoethanol
10µM SB431542
100nM LDN193189
(+10µM Y27632 overnight only, then remove)

Neural Maintenance Media:

1:1 DMEM-F12: Neurobasal
1x N2 supplement (with 20µg/ml final Insulin-Zinc concentration)
0.05x B27 supplement without Vitamin A
2mM Glutamax
0.1mM Beta-mercaptoethanol
(4F):
10ng/ml FGF2
3µM CHIR99021
1µM SB431542 (or FST 50ng/mL)
100nM LDN193189 (or NOGGIN 50ng/mL)
(6F):
100nM K02288
100nM AKTiVIII
75nM MK2006 (AKT1/2/3i)
1-2µM XAV939
(+10µM Y27632 overnight only, then remove – can omit after 3rd passage as cNESCs will survive as single cells)

Neural Differentiation I Media:

1:1 DMEM-F12: Neurobasal
0.5x N2 supplement (with 20µg/ml final Insulin-Zinc concentration) 0.5x B27 supplement with Vitamin A
2mM Glutamax
0.1mM Beta-mercaptoethanol
*no inhibitors or growth factors!

Neural Differentiation II Media:

1:1 DMEM-F12: Neurobasal
0.5x N2 supplement (with 20µg/ml final Insulin-Zinc concentration) 1x B27 supplement with Vitamin A
2mM Glutamax
0.1mM Beta-mercaptoethanol
10ng/mL BDNF
10µM Forskolin

Homemade N2 supplement (100x):

Concentration
Progesterone 0.002016mg/ml
Putrescine 1.629mg/ml
Selenium 0.00305mM
Bovine Albumin Fraction V 0.75%
Apo-transferrin 10mg/ml

Supplementary Table 1
cNESC culture protocol Details of derivation, maintenance and differentiation of cNESCs. List of components of cell culture media.

			CNES markers			Differentiation markers			
**hPSC line**	**cNES cell line**	**Factors**	**FOXG1**	**PAX6**	**OTX2**	**TBR1**	**BCL11B**	**SATB2**	**GFAP**
**H1**	**H1A**	4F	-	+	-	-	+	+	+
**H9**	**H9A**	4F	-	+	-	-	+	+	+
**CA1**	**CA1**	4F	-	+	-	-	+	+	+
**1.53E**	**1.53E**	4F	-	+	-	-	+	+	+
**H1**	**H1JA**	6F	+	low	+	+	+	+	+
**CA1**	**CA1J**	6F	+	-	+	+	+	+	+
**CTRL**	**CTRL**	6F	+	low	+	+	+	+	+
**SHEF6**	**S6**	6F	+	+	-	+	+	+	+
**H1**	**H1JA-B4**	6F	+	+	+	+	-	-	-
**H1**	**H1JA-C4**	6F	+	+	+	+	+	+	+
**H1**	**H1JA-D4**	6F	+	+	+	+	-	-	-
**H1**	**H1JA-E4**	6F	+	+	+	+	-	-	-
**H1**	**H1JA-D2**	6F	low	+	low	+	NA	NA	NA
**H9**	**H9J**	6F	+	+	-	+	+	+	+
**SHEF6**	**S6cA1**	6F	+	+	-	+	+	+	NA
**SHEF6**	**S6cA2**	6F	+	+	-	+	+	+	NA
**SHEF6**	**S6cA3**	6F	+	+	-	+	+	+	NA
**SHEF6**	**S6cA4**	6F	+	+	-	+	+	+	NA
**SHEF6**	**S6cA5**	6F	+	+	-	+	+	+	NA
**SHEF6**	**S6cA6**	6F	+	+	-	+	+	+	NA
**SHEF6**	**S6cA7**	6F	+	+	-	+	+	+	NA
**SHEF6**	**S6cA8**	6F	+	+	-	+	+	+	NA
**SHEF6**	**S6cA9**	6F	+	+	-	+	+	+	NA
**SHEF6**	**S6cA10**	6F	+	+	-	+	+	+	NA
**SHEF6**	**S6cA11**	6F	+	+	-	+	+	+	NA
**SHEF6**	**S6cA12**	6F	+	+	-	+	+	+	NA
**SHEF6**	**S6cB1**	6F	+	+	-	+	+	+	NA
**SHEF6**	**S6cB2**	6F	+	+	-	+	+	+	NA
**SHEF6**	**S6cB3**	6F	+	+	-	+	+	+	NA
**SHEF6**	**S6cB4**	6F	+	+	-	+	+	+	NA
**SHEF6**	**S6cB5**	6F	+	+	-	+	+	+	NA
**SHEF6**	**S6cB6**	6F	+	+	-	+	+	+	NA
**SHEF6**	**S6cB7**	6F	+	+	-	+	+	+	NA
**SHEF6**	**S6cB8**	6F	+	+	-	+	+	+	NA
**SHEF6**	**S6cB9**	6F	+	+	-	+	+	+	NA
**SHEF6**	**S6cB10**	6F	+	+	-	+	+	+	NA
**SHEF6**	**S6cB11**	6F	+	+	-	+	+	+	NA
**SHEF6**	**S6cB12**	6F	+	+	-	+	+	+	NA
**SHEF6**	**S6cC1**	6F	+	+	-	+	+	+	NA
**SHEF6**	**S6cC2**	6F	+	+	-	+	+	+	NA
**SHEF6**	**S6cC3**	6F	+	+	-	+	+	+	NA
**SHEF6**	**S6cC4**	6F	+	+	-	+	+	+	NA
**SHEF6**	**S6cC5**	6F	+	+	-	+	+	+	NA
**SHEF6**	**S6cC6**	6F	+	+	-	+	+	+	NA
**SHEF6**	**S6cC7**	6F	+	+	-	+	+	+	NA
**SHEF6**	**S6cC8**	6F	+	+	-	+	+	+	NA
**SHEF6**	**S6cC9**	6F	+	+	-	+	+	+	NA
**SHEF6**	**S6cC10**	6F	+	+	-	+	+	+	NA
**SHEF6**	**S6cC11**	6F	+	+	-	+	+	+	NA
**SHEF6**	**S6cC12**	6F	+	+	-	+	+	+	NA
**SHEF6**	**S6cD1**	6F	+	+	-	+	+	+	NA
**SHEF6**	**S6cD2**	6F	+	+	-	+	+	+	NA
**SHEF6**	**S6cD3**	6F	+	+	-	+	+	+	NA
**SHEF6**	**S6cD4**	6F	+	+	-	+	+	+	NA
**SHEF6**	**S6cD5**	6F	+	+	-	+	+	+	NA
**SHEF6**	**S6cD6**	6F	+	+	-	+	+	+	NA
**SHEF6**	**S6cD7**	6F	+	+	-	+	+	+	NA
**SHEF6**	**S6cD8**	6F	+	+	-	+	+	+	NA
**SHEF6**	**S6cD9**	6F	+	+	-	+	+	+	NA
**SHEF6**	**S6cD10**	6F	+	+	-	+	+	+	NA
**SHEF6**	**S6cD11**	6F	+	+	-	+	+	+	NA
**SHEF6**	**S6cD12**	6F	+	+	-	+	+	+	NA

Supplementary Table 2
Details of cNESC lines used in the study.

REAGENT or RESOURCE	SOURCE	IDENTIFIER			
Antibodies			Clone name	Working Dilution	Validation
a-TUBULIN	Cell Signaling	2144		1:500	By company
BRN2	SANTA CRUZ	sc-6029		1:200	By company
BCL11B	Abcam	ab18465	25B6	1:100	By company
CUX1	SANTA CRUZ	sc-13024		1:200	By company
FOXG1	Abcam	ab18259		1:100	Negative control: pluripotent stem cells
GAD67	Abcam	ab26116	K-87	1:100	By company
GFAP	DAKO	Z0334		1:500	By company
GFP	Abcam	ab13970		1:1000	By company
HUNU	Millipore	MAB1281		1:500	Negative control: mouse brain tissue
KI67	ThermoFisher	MA5-14520	SP6	1:200	By company
MAP2ab	Sigma-Aldrich	M1406	HM-2	1:1000	By company
NANOG	ReproCell	RCAB003P		1:200	By company
NESTIN	GeneTex	GTX30670	10C2	1:400	By company
NCAM	Abcam	ab75813	EP2567Y	1:200	By company
NEUN	Millipore	MAB377	A60	1:500	By company
O4	Millipore	MAB345	O4	1:200	By company
POU5F1	SANTA CRUZ	sc-5279	C-10	1:200	By company
OTX1/2	Abcam	ab21990		1:100	By company
PAX6	Covance	PRB-278P-100		1:100	Negative control: pluripotent stem cells
pERK	Cell Signaling	9101		1:1000	By company
pFOXO1	Cell Signaling	9461		1:1000	By company
pP70S6K	Cell Signaling	9204		1:1000	By company
pSHC1	Cell Signaling	2434		1:1000	By company
p21 Waf1/Cip1	Cell Signaling	2947	12D1	1:100	By company
SATB2	Abcam	ab51502	SATBA4B10	1:100	By company
SOX1	Cell Signaling	4194S		1:400	Negative control: pluripotent stem cells
SOX2	RnD Systems	MAB2018	245610	1:200	By company
SYNAPTOPHYSIN/SYP	DAKO	IR660	DAK-SYNAP	1:500	By company
TBR1	Robert Hevner	NA		1:1000	Negative control: pluripotent stem cells, verified by donor lab
TBR1	Abcam	ab31940		1:200	Negative control: pluripotent stem cells
tERK	SANTA CRUZ	sc-93		1:1000	By company
tSHC1	BD	610879	30/SHC	1:1000	By company
STEM121	Takara	ab-121	SC121	1:500	Negative control: mouse brain tissue
TUBB3	Sigma-Aldrich	SAB4700544	TU-20	1:8000	By company
TUBB3	Sigma-Aldrich	T3952		1:2000	By company
VGAT	Synaptic system	131 003	Gp117G4	1:1000	By company, knock-out mouse
VGLUT1	Synaptic system	135 303	68B7	1:1000	By company, knock-out mouse

Goat Anti-Chicken IgY H&L (Alexa Fluor 488)	Abcam	ab150169		1:500	Negative control: cell or tissue without chicken IgY labelling
Goat anti-Mouse IgG (H+L) (Alexa Fluor 647)	Thermo Fisher Scientific	A21236		1:500	Negative control: cell or tissue without mouse IgG labelling
Goat anti-Mouse IgG (H+L) (Alexa Fluor 568)	Thermo Fisher Scientific	A-11004		1:500	Negative control: cell or tissue without mouse IgG labelling
Goat anti-Mouse IgG (H+L) (Alexa Fluor 488)	Thermo Fisher Scientific	A-11001		1:500	Negative control: cell or tissue without mouse IgG labelling
Goat anti-Rabbit IgG (H+L) (Alexa Fluor 647)	Thermo Fisher Scientific	A-21245		1:500	Negative control: cell or tissue without rabbit IgG labelling
Goat anti-Rabbit IgG (H+L) (Alexa Fluor 568)	Thermo Fisher Scientific	A-11011		1:500	Negative control: cell or tissue without rabbit IgG labelling
Goat anti-Rabbit IgG (H+L) (Alexa Fluor 488)	Thermo Fisher Scientific	A-11008		1:500	Negative control: cell or tissue without rabbit IgG labelling
Donkey anti-Goat IgG (H+L) (Alexa Fluor 488)	Thermo Fisher Scientific	A-11055		1:500	Negative control: cell or tissue without rabbit IgG labelling
Goat anti-Mouse IgG (H+L) (Alexa Fluor 488)	Thermo Fisher Scientific	A-10680		1:500	Negative control: cell or tissue without mouse IgM labelling
Chemicals, Peptides, and Recombinant Proteins					
2-mercaptoethanol	Sigma-Aldrich	M3148			
Accutase	Stem Cell Technologies	7920			
ACTIVIN A	Peprotech	120-14			
Aggrewell 800	Stem Cell Technologies	34811			
AKTiVIII	Calbiochem	124018			
Alamar blue	ThermoFisher	DAL1025			
B27	Life Technologies	17504044			
B27-VA	Life Technologies	12587010			
BDNF	Peprotech	450-02			
BMP4	Peprotech	120-05			
BSA fV	Life Technologies	15260037			
CHIR99021	Selleckchem	S2924			
Cyclopamine-KAAD	Calbiochem	239804			
DAPT	Selleckchem	S2215			
DMEM-F12	Life Technologies	21331-020			
EGF	Peprotech	100-47			
FGF2	Peprotech	100-18B			
FOLLISTATIN	Peprotech	120-13			
Glutamax	Life Technologies	35050061			
Insulin-Zinc	Life Technologies	12585014			
K02288	Selleckchem	S7359			
KSR	Life Technologies	10828028			

Laminin	Sigma-Aldrich	L2020			
LDN193189	Selleckchem	S2618			
MK2206	Selleckchem	S1078			
mTESR	Stem Cell Technologies	5850			
N2	Homemade	NA			
NEAA	Life Technologies	11140050			
Neurobasal	Life Technologies	21103-049			
NOGGIN	Peprotech	120-10C			
PD0325901	Selleckchem	S1036			
PD153035	Selleckchem	S1079			
PD173074	Selleckchem	S1264			
PI-103	Calbiochem	528100			
Poly-ornithine	Sigma-Aldrich	P4957			
Purmorphamine	Selleckchem	S3042			
SAG	Selleckchem	S7779			
SB431542	Selleckchem	S1067			
SB505124	Selleckchem	S2186			
TGFβ1	Peprotech	100-21			
Wortmannin	Calbiochem	681675			
XAV939	Selleckchem	S1180			
Progesterone	Sigma-Aldrich	P8783			
Putrescine dihydrochloride	Sigma-Aldrich	P5780			
Sodium selenite	Sigma-Aldrich	S5261			
Bovine Albumin Fraction V	Gibco	15260037			
apo-Transferrin human	Sigma-Aldrich	T1147			
Y27632	Selleckchem	S1049			
Experimental Models: Cell Lines					
Human: Passage 32 H1 ES cells	WiCell	WAe001-A			
Human: Passage 33 H9 ES cells	WiCell	WAe009-A			
Human: Passage 29 CA1 ES cells	Nagy laboratory				
Human: Passage 34 SHEF6 ES cells	UK Stem Cell Bank (UKSCB)	R-05-031			
Human: Passage 25 1.53 iPS cells	Hussein, SM. et al. 2014				
Human: Passage 16 CTRL iPS cells	Vallier, L. et al. 2009				
Human: Passage 25 CB660 NS cells	Sun, Y. et al. 2018				
Oligonucleotides					
hOCT4t	CCCTCGTGCAGGCCCGAAAG	CCCAAGCTGCTGGGCGATGT			
hNESTIN	GGCGCACCTCAAGATGTCC	CTTGGGGTCCTGAAAGCTG			
hFOXG1	GCCACAATCTGTCCCTCAACA	CGGGTCCAGCATCCAGTAG			
hOTX2	CAACCGCCTTACGCAGTCAA	GGGGTGCAGCAAGTCCATAC			

hFOXA2	GGAGCAGCTACTATGCAGAGC	CGTGTTCATGCCGTTCATCC			
hEN1	GAGCGCAGGGCACCAAATA	AATAACGTGTGCAGTACACCC			
hHOXB1	AGGAGACGGAGGCTATTTTCA	GTCTGCTCGTTCCCATAAGGG			
hPAX6	ATGTGTGAGTAAAATTCTGGGCA	GCTTACAACTTCTGGAGTCGCTA			
hEMX2	CGGCACTCAGCTACGCTAAC	CAAGTCCGGGTTGGAGTAGAC			
hLHX6	TGAGAGTCAGGTACAGTGCG	GCCCATCCATATCGGCTTTGA			
hNKX2.1	AGCACACGACTCCGTTCTC	GCCCACTTTCTTGTAGCTTTCC			
hGLI1	ACAGAAGGACTGTCTGGCCC	AGCTGTTGGTCTCTCTGGC			
hPTCH1	CCACAGAAAACCCCGTCTTC	GGTTCGAGGGTGGGTGATG			
hHHIP	ATGGTGGGTTGTGCTTTCC	AGTTGTGTTTGTGCTTTCTGCT			
hGAPDH	CATGAGAAGTATGACAACAGCCT	AGTCCTTCCACGATACCAAAGT			
hEMX1	AGGTGAAGGTGTGGTTCCAG	AGTCATTGGAGGTGACATCG			
hLMX1A	GCAAAGGGGACTATGAGAAGGA	CGTTTGGGGCGCTTATGGT			
hDLL1	GATTCTCCTGATGACCTCGCA	TCCGTAGTAGTGTTCGTCACA			
hNANOG	CCTTGGCTGCCGTCTCTGGC	AGCAAAGCCTCCCAATCCCAAACA			
hNGN2	CACCACAACACACGAGACC	ACTCCAAGGTCTCGGATTTGAC			
hHES1	TCAACACGACACCGGATAAAC	GCCGCGAGCTATCTTTCTTCA			
hSHH	CTCCAGAAACTCCGAGCGAT	CCCTCGTAGTGCAGAGACTC			
hHES5	CGGGCACATTTGCCTTTTGT	GTCCGGGGTGATCACTGTTT			
hNGN1	GCTCTCTGACCCCAGTAGC	GCGTTGTGTGGAGCAAGTC			
hDLL3	CACTCCCGGATGCACTCAAC	GATTCCAATCTACGGACGAGC			
hDLX2	ACGCTCCCTATGGAACCAGTT	TCCGAATTTCAGGCTCAAGGT			
hHES3	GCACGCATCAATGTGTCACTG	CCTTCTCCAATTTGCGCTTCC			
hASCL1	TCTTCGCCCGAACTGATGC	CAAAGCCCAGGTTGACCAACT			
hAXIN2	AGTGTGAGGTCCACGGAAAC	CTGGTGCAAAGACATAGCCA			
hLEF1	TGGATCTCTTTCTCCACCCA	CACTGTAAGTGATGAGGGGG			
hGBX2	GGTGCAGGTGAAAATCTGGT	CCTGTCTTGGAATTGGCATT			
hYWHAZ	CCGCCAGGACAAACCAGTAT	ACTTTTGGTACATTGTGGCTTCAA			
Software and Algorithms					
Volocity 6.1.1	Perkin Elmer				
MeV 4.8		https://sourceforge.net/projects/mev-tm4/files/mev-tm4/MeV%204.8.1/			

Graphpad Prism 8		https://www.graphpad.com/scientific-software/prism/			
Oligo (v1.34.2)	Irizarry et al., 2003	https://bioc.ism.ac.jp/packages/3.2/bioc/bin/macosx/mavericks/contrib/3.2/oligo_1.34.2.tgz			
Limma (v3.26.9)	Phipson et al., 2016				
Stats (v3.2.2)		http://www.rdocumentation.org/badges/version/stats			
Pathview (v1.10.1)	Luo and Brouwer, 2013	http://r-forge.r-project.org/projects/pathview/			
ClusterProfiler (v2.4.3)	Yu et al., 2012	https://bioc.ism.ac.jp/packages/3.2/bioc/bin/macosx/mavericks/contrib/3.2/clusterProfiler_2.4.3.tgz			
Gplots (v3.0.1.1)		https://cran.r-project.org/src/contrib/gplots_3.0.1.1.tar.gz			
Guppy v4.2.2		https://github.com/a-slide/singularity/blob/main/ont_guppy/ont_guppy:CPU-4.2.2.srf			
Minimap2		https://github.com/lh3/minimap2			
EdgeR R-package (v3.12.1)		https://bioconductor.org/packages/release/bioc/html/edgeR.html			
STAR aligner v2.7.5a		https://github.com/alexdobin/STAR/releases/tag/2.7.5a			
Samtools v1.11		https://github.com/samtools/samtools/releases/tag/1.11			
ComplexHeatmaps R package		https://bioconductor.org/packages/release/bioc/html/ComplexHeatmap.html			
pCLAMP10, Clampex, Clampfit		https://www.moleculardevices.com/products/axon-patch-clamp-system/acquisition-and-analysis-software/pclamp-software-suite			
Leica LAS AF		https://www.leica-microsystems.com/			
Zeiss Zen 3		https://www.zeiss.com			
MATLAB r2018b		https://uk.mathworks.com			

Supplementary Table 3

Details of reagents and resources used in the study.
