# Supplementary material for: Signal requirement for cortical potential of transplantable human neuroepithelial stem cells
Source: Nat Commun. 2022 May 23;13:2844. doi: 10.1038/s41467-022-29839-8 (PMC9126949; doi:10.1038/s41467-022-29839-8)
Supplement: Supplementary file 3 — Reporting Summary [file 41467_2022_29839_MOESM3_ESM.pdf]

Corresponding author(s): Andras Nagy PhD, Balazs Varga PhD

Last updated by author(s): 23/Feb/2022

Reporting Summary

Nature Portfolio wishes to improve the reproducibility of the work that we publish. This form provides structure for consistency and transparency in reporting. For further information on Nature Portfolio policies, see our Editorial Policies and the Editorial Policy Checklist.

Statistics

For all statistical analyses, confirm that the following items are present in the figure legend, table legend, main text, or Methods section.

n/a	Confirmed	
☐	☒	The exact sample size (*n*) for each experimental group/condition, given as a discrete number and unit of measurement
☐	☒	A statement on whether measurements were taken from distinct samples or whether the same sample was measured repeatedly
☐	☒	The statistical test(s) used AND whether they are one- or two-sided<br>*Only common tests should be described solely by name; describe more complex techniques in the Methods section.*
☐	☒	A description of all covariates tested
☐	☒	A description of any assumptions or corrections, such as tests of normality and adjustment for multiple comparisons
☐	☒	A full description of the statistical parameters including central tendency (e.g. means) or other basic estimates (e.g. regression coefficient) AND variation (e.g. standard deviation) or associated estimates of uncertainty (e.g. confidence intervals)
☐	☒	For null hypothesis testing, the test statistic (e.g. *F*, *t*, *r*) with confidence intervals, effect sizes, degrees of freedom and *P* value noted<br>*Give P values as exact values whenever suitable.*
☒	☐	For Bayesian analysis, information on the choice of priors and Markov chain Monte Carlo settings
☒	☐	For hierarchical and complex designs, identification of the appropriate level for tests and full reporting of outcomes
☒	☐	Estimates of effect sizes (e.g. Cohen's *d*, Pearson's *r*), indicating how they were calculated

Our web collection on statistics for biologists contains articles on many of the points above.

Software and code

Policy information about availability of computer code

Data collection	Imaging data was collected with Volocity 6.1.1, Zeiss Zen 3 and Leica LAF softwares. QPCR data was collected with Bio-RAD CFX384. RNA-sequencing data was collected with MinKNOW software v19. Electrophyisological recordings were processed with Clampex 10 or 11.0.3, pClamp10.3 or 10.7 software.
Data analysis	Volocity 6.1.1 Perkin Elmer<br>MeV 4.8 https://sourceforge.net/projects/mev-tm4/files/mev-tm4/MeV%204.8.1/<br>Graphpad Prism 8 https://www.graphpad.com/scientific-software/prism/<br>Excel 2016,2018, 365<br>Oligo (v1.34.2) Irizarry et al., 2003<br>https://bioc.ism.ac.jp/packages/3.2/bioc/bin/macosx/mavericks/contrib/3.2/oligo_1.34.2.tgz<br>Limma (v3.26.9) Phipson et al., 2016<br>Stats (v3.2.2) http://www.rdocumentation.org/badges/version/stats<br>Pathview (v1.10.1) Luo and Brouwer, 2013<br>http://r-forge.r-project.org/projects/pathview/<br>ClusterProfiler (v2.4.3) Yu et al., 2012<br>https://bioc.ism.ac.jp/packages/3.2/bioc/bin/macosx/mavericks/contrib/3.2/clusterProfiler_2.4.3.tgz<br>Gplots (v3.0.1.1) https://cran.r-project.org/src/contrib/gplots_3.0.1.1.tar.gz<br>Guppy v.4.2.2 https://github.com/a-slide/singularity/blob/main/ont_guppy/ont_guppy:CPU-4.2.2.srf<br>Minimap2 https://github.com/lh3/minimap2<br>EdgeR R-package (v3.12.1) https://bioconductor.org/packages/release/bioc/html/edgeR.html<br>STAR aligner v2.7.5a https://github.com/alexdobin/STAR/releases/tag/2.7.5a<br>Samtools v1.11 https://github.com/samtools/samtools/releases/tag/1.11

ComplexHeatmaps R package https://bioconductor.org/packages/release/bioc/html/ComplexHeatmap.html
Clampfit 10 or 11.0.3 https://www.moleculardevices.com/products/axon-patch-clamp-system/acquisition-and-analysis-software/pclamp-software-suite
MATLAB r2018b

For manuscripts utilizing custom algorithms or software that are central to the research but not yet described in published literature, software must be made available to editors and reviewers. We strongly encourage code deposition in a community repository (e.g. GitHub). See the Nature Portfolio guidelines for submitting code & software for further information.

Data

Policy information about availability of data

All manuscripts must include a data availability statement. This statement should provide the following information, where applicable:

- Accession codes, unique identifiers, or web links for publicly available datasets
- A description of any restrictions on data availability
- For clinical datasets or third party data, please ensure that the statement adheres to our policy

Data generated in this study were deposited to the Gene Expression Omnibus. Microarray and long-read RNA sequencing data are available under accession numbers GSE185258 and GSE184081, respectively. Publicly available datasets used in this study can be accessed under accession numbers GSE107122 and GSE81475. The KEGG and DAVID databases were used for gene ontology enrichment analysis. The data are provided as Source Data files.

Field-specific reporting

Please select the one below that is the best fit for your research. If you are not sure, read the appropriate sections before making your selection.

[x] Life sciences [ ] Behavioural & social sciences [ ] Ecological, evolutionary & environmental sciences

For a reference copy of the document with all sections, see nature.com/documents/nr-reporting-summary-flat.pdf

Life sciences study design

All studies must disclose on these points even when the disclosure is negative.

Sample size	We did not predetermine the sample size. We used at least 3 independent iPSC derived cell lines for experiments that involved paired measurements. For descriptive experiments like electrophysiology of neurons we used the cell line that was included in all other measurements. The sample sizes and number of repeats are defined in each figure legends.
Data exclusions	No data was excluded from the analysis.
Replication	Each experiment was repeated independently as indicated. Sample sizes and number of repeats are defined in each figure legends. Kinase inhibitor activity was not repeatedly validated by western blot.
Randomization	For comparative experiments we used the same cell lines, and measured all experimental conditions in all cell lines, therefore randomization was not necessary.
Blinding	The experimental samples for QPCR and Western blot were identified with codes by BV and analyzed blindly by HJ and EL. The cell culture (cell cloning, colony formation) experiments were done by BV alone so blinding was not possible. Descriptive experiments such as cell transplantation, electrophysiology were done without blinding. Image analysis was done with automated protocols of Volocity software after background correction.

Reporting for specific materials, systems and methods

We require information from authors about some types of materials, experimental systems and methods used in many studies. Here, indicate whether each material, system or method listed is relevant to your study. If you are not sure if a list item applies to your research, read the appropriate section before selecting a response.

Materials & experimental systems

n/a	Involved in the study	
[ ]	[x]	Antibodies
[ ]	[x]	Eukaryotic cell lines
[x]	[ ]	Palaeontology and archaeology
[ ]	[x]	Animals and other organisms
[x]	[ ]	Human research participants
[x]	[ ]	Clinical data
[x]	[ ]	Dual use research of concern

Methods

n/a	Involved in the study	
[x]	[ ]	ChIP-seq
[x]	[ ]	Flow cytometry
[x]	[ ]	MRI-based neuroimaging

Antibodies

Antibodies used

Antibody target, Company, catalogue number, clone code if applicable, dilution used, validation:

a-TUBULIN, Cell Signaling, 2144, 1:1000, By company
BRN2, SANTA CRUZ, sc-6029, 1:200, By company
BCL11B, Abcam, ab18465, clone 25B6, 1:100, By company
CUX1, SANTA CRUZ, sc-13024, 1:200, By company
FOXG1, Abcam, ab18259, 1:100, Negative control: pluripotent stem cells
GAD67, Abcam, ab26116, K-87, 1:100, By company
GFAP, DAKO, Z0334, 1:500, By company
GFP, Abcam, ab13970, 1:1000, By company
HUNU, Millipore, MAB1281, 1:500, Negative control: mouse brain tissue
KI67, ThermoFisher, MA5-14520, SP6, 1:200, By company
MAP2ab, Sigma, M1406, HM-2, 1:1000, By company
NANOG, ReproCell, RCAB003P, 1:200, By company
NESTIN, GeneTex, GTX30670, 10C2, 1:400, By company
NCAM, Abcam, ab75813, EP2567Y, 1:200, By company
NEUN, Millipore, MAB377, A60, 1:500, By company
O4, Millipore, MAB345, O4, 1:200, By company
POU5F1, SANTA CRUZ, sc-5279, C-10, 1:200, By company
OTX1/2, Abcam, ab21990, 1:100, By company
PAX6, Covance, PRB-278P-100, 1:100, By company
pERK, Cell Signaling, 9101, 1:1000, By company
pFOXO1, Cell Signaling, 9461, 1:1000, By company
pP70S6K, Cell Signaling, 9204, 1:1000, By company
pSHC1, Cell Signaling, 2434, 1:1000, By company
p21 Waf1/Cip1, Cell Signaling, 2947, 12D1, 1:100 By company
SATB2, Abcam, ab51502, SATBA4B10, 1:100, By company
SOX1, Cell Signaling, 4194S, 1:400, Negative control: pluripotent stem cells
SOX2, RnD Systems, MAB2018, 245610, 1:200 By company
SYNAPTOPHYSIN/SYP, DAKO, IR660, DAK-SYNAP, 1:500, By company
TBR1, Robert Hevner NA, 1:1000, Negative control: pluripotent stem cells, verified by donor lab
TBR1, Abcam, ab31940, 1:200, Negative control: pluripotent stem cells
tERK, SANTA CRUZ, sc-93, 1:1000, By company
tSHC1, BD 610879, 30/SHC, 1:1000, By company
STEM121, Takara, ab-121, SC121, 1:500, Negative control: mouse brain tissue
TUBB3, Sigma, SAB4700544, TU-20, 1:8000, By company
TUBB3, Sigma, T3952, 1:2000, By company
VGAT, Synaptic system, 131 003, Gp117G4, 1:1000, By company, knock-out mouse
VGLUT1, Synaptic system, 135 303, 68B7, 1:1000, By company, knock-out mouse
Goat Anti-Chicken IgY H&L (Alexa Fluor 488) Abcam ab150169 1:500 Negative control: cell or tissue without chicken IgY labelling
Goat anti-Mouse IgG (H+L) (Alexa Fluor 647) Thermo Fisher Scientific A21236 1:500 Negative control: cell or tissue without mouse IgG labelling
Goat anti-Mouse IgG (H+L) (Alexa Fluor 568) Thermo Fisher Scientific A-11004 1:500 Negative control: cell or tissue without mouse IgG labelling
Goat anti-Mouse IgG (H+L) (Alexa Fluor 488) Thermo Fisher Scientific A-11001 1:500 Negative control: cell or tissue without mouse IgG labelling
Goat anti-Rabbit IgG (H+L) (Alexa Fluor 647) Thermo Fisher Scientific A-21245 1:500 Negative control: cell or tissue without rabbit IgG labelling
Goat anti-Rabbit IgG (H+L) (Alexa Fluor 568) Thermo Fisher Scientific A-11011 1:500 Negative control: cell or tissue without rabbit IgG labelling
Goat anti-Rabbit IgG (H+L) (Alexa Fluor 488) Thermo Fisher Scientific A-11008 1:500 Negative control: cell or tissue without rabbit IgG labelling
Donkey anti-Goat IgG (H+L) (Alexa Fluor 488) Thermo Fisher Scientific A-11055 1:500 Negative control: cell or tissue without rabbit IgG labelling
Goat anti-Mouse IgG (H+L) (Alexa Fluor 488) Thermo Fisher Scientific A-10680 1:500 Negative control: cell or tissue without mouse IgM labelling

Validation

Antibodies were used as indicated on the product datasheet. Negative controls -cells that do not express the mRNA coding the target epitope- like pluripotent human stem cells were used for neural specific epitopes. Neural cells or fibroblast was used as negative controls for pluripotent cell specific epitopes.

Eukaryotic cell lines

Policy information about cell lines

Cell line source(s)	H1 (WAe001-A, WiCell), H9 (WAe009-A,WiCell), CA1 (Mount Sinai Hospital, Canada), SHEF6 (R-05-031,UK Stem Cell Bank), CTRL-2429 (University of Cambridge), 1.53E (Mount Sinai Hospital, Canada)
Authentication	The cell lines were not authenticated.
Mycoplasma contamination	All cell lines were repeatedly tested negative for mycoplasma contamination.
Commonly misidentified lines (See ICLAC register)	No commonly misidentified cell lines were used in the study.

Animals and other organisms

Policy information about studies involving animals; ARRIVE guidelines recommended for reporting animal research

Laboratory animals	NOD.Cg-Prkdc-scid Il2rgtm1Wjl/SzJ (Strain #:005557), females 8-10 weeks old, 12 animals.
Wild animals	*Provide details on animals observed in or captured in the field; report species, sex and age where possible. Describe how animals were caught and transported and what happened to captive animals after the study (if killed, explain why and describe method; if released, say where and when) OR state that the study did not involve wild animals.*
Field-collected samples	*For laboratory work with field-collected samples, describe all relevant parameters such as housing, maintenance, temperature, photoperiod and end-of-experiment protocol OR state that the study did not involve samples collected from the field.*
Ethics oversight	All human stem cell work complied with the human stem cell guidance and was approved by the Stem Cell Oversight Committee of Canada and the Human Tissue Authority of United Kingdom, all animal work complied with regulations in Canada and United Kingdom and was approved by Mount Sinai Hospital Research Ethical board (Canada) and the Home office (UK).

Note that full information on the approval of the study protocol must also be provided in the manuscript.
